# Deciphering grain-size reduction as a driver of mid-lithosphere discontinuity formation

**DOI:** 10.1126/sciadv.aed4229

**Published:** 2026-04-24

**Authors:** Mingqi Liu, Taras V. Gerya, Zhong-Hai Li, Ling Chen, James A. D. Connolly

**Affiliations:** ^1^ Department of Earth and Planetary Sciences, ETH Zürich, Sonneggstrasse 5, CH-8092 Zürich, Switzerland.; ^2^ State Key Laboratory of Earth System Numerical Modeling and Application, College of Earth and Planetary Sciences, University of Chinese Academy of Sciences, Beijing 101408, China.; ^3^ State Key Laboratory of Lithospheric and Environmental Coevolution, Institute of Geology and Geophysics, Chinese Academy of Sciences, Beijing 100029, China.; ^4^ College of Earth and Planetary Sciences, University of Chinese Academy of Sciences, Beijing 100049, China.; ^5^ Centre for Earth Sciences, Indian Institute of Science, Bengaluru, Karnataka 560012, India.

## Abstract

The mid-lithosphere discontinuity (MLD) is a fundamental yet enigmatic seismic boundary within Earth’s lithosphere, whose origin remains debated despite decades of study. Using thermomechanical models with self-consistent grain-size evolution, we propose a previously unrecognized mechanism for MLD formation. Plate deformation induces pronounced grain-size reduction within the brittle-ductile transition, generating permeability barriers that trap ascending fluids. These fluid-rich layers reproduce the observed seismic velocity drops. Their depth scales with lithospheric thickness and thermal structure and is fine-tuned by rheology evolution through brittle-ductile partitioning. The interplay between grain-size reduction and fluid accumulation imparts dual mechanical behavior to the MLD, stabilizing it within cold, ancient lithosphere or enabling localized failure under thermal perturbation. This framework identifies grain-scale processes as the primary control on MLD formation and links mantle-lithosphere coupling to planetary lithospheric evolution.

## Teaser

Deformation-driven grain-size reduction traps fluids in Earth’s lithosphere, forming a major seismic boundary.

## INTRODUCTION

Decades of geophysical, petrological, and geochemical studies reveal a highly heterogeneous lithospheric mantle, characterized by layered structures that vary systematically across tectonic settings (*1*–*5*). Among these structures, two seismic discontinuities stand out as globally recognized: the lithosphere-asthenosphere boundary (LAB) and the mid-lithosphere discontinuity (MLD) (*5*–*9*). The LAB demarcates the base of rigid tectonic plates, separating them from the underlying low-viscosity asthenosphere (*10*). This transition, attributed to partial melting, hydration, and/or thermal gradients, is critical for facilitating plate motion and maintaining plate integrity (*11*–*14*). In contrast, the origin and geodynamic significance of the ubiquitous MLD, typically manifesting as a sharp reduction of seismic shear wave velocity (*V*_s_) within cratonic and oceanic lithosphere (*2*, *15*, *16*), remain enigmatic. Critically, the physical mechanisms controlling the MLD’s depth distribution, sharpness, and rheological implications are poorly constrained, presenting a major unresolved challenge in plate tectonics and lithospheric geodynamics.

**Fig. 1.**
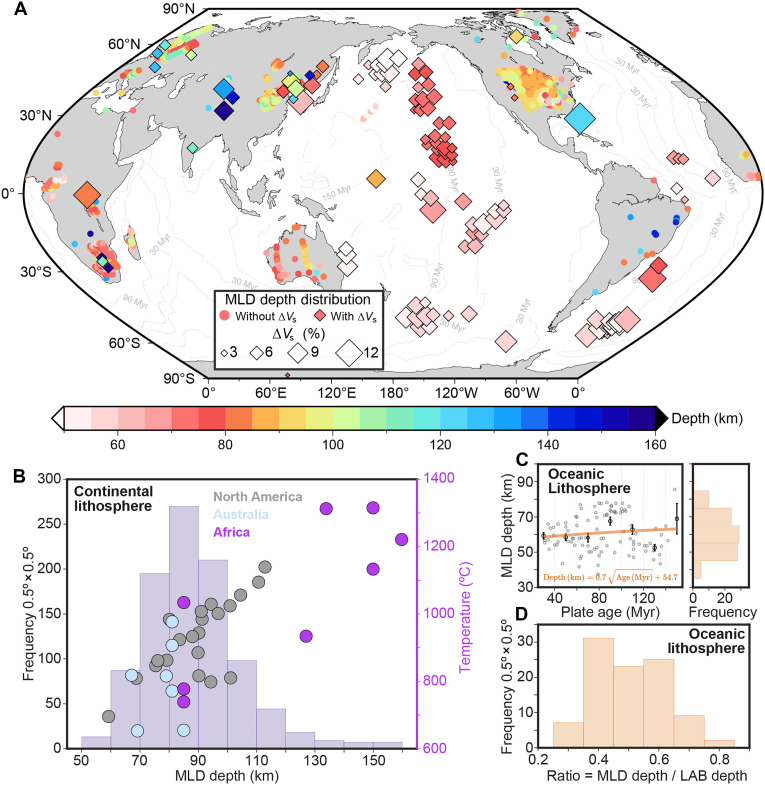
Compilation of global MLD observations. (**A**) Depth to the discontinuity within the oceanic and continental mantle lithosphere. Circles and squares mark MLD locations reported from previous studies (*18*, *19*, *32*, *84*). The size of the squares corresponds to the magnitude of the shear wave velocity (*V*_s_) drop. Light gray lines represent the age contour of oceanic plates (*85*). (**B**) Spatial frequency of observed discontinuity depths in the continental lithosphere. Data points indicate temperatures at MLD depths within the North American, Australian, and African plates (*15*). (**C**) Shallow oceanic discontinuities (<100 km) in the Pacific and Atlantic [plate age > 30 million years ago (Ma)] (*18*–*20*). Left: MLD depth versus seafloor age (gray circles indicate individual observations; error bars show 20-Myr binned means ±1 SE); the orange line denotes the fitted age-MLD depth scaling. Right: Frequency distribution binned at 0.5° × 0.5°. (**D**) Frequency distribution of the ratio between shallow discontinuity depth and the thermally defined LAB depth at a constant mantle potential temperature (based on the plate cooling model with a plate thickness of 125 km) within the oceanic lithosphere (*41*, *42*). Myr, million years.

A diverse suite of seismic techniques consistently images the MLD globally (Fig. 1). Key observations include long-range active-source profiles (*17*, *18*), SS precursors (*19*, *20*), teleseismic P and S receiver functions (*5*, *21*–*24*), anisotropy models from joint surface/body-wave inversions (*25*), and surface-wave tomography using both teleseismic and ambient noise (*26*). Notably, continental MLDs exhibit sharp *V*_s_ reductions (2 to 10%) over narrow depth intervals (≤30 to 40 km) (*2*, *27*) at subsolidus temperatures (600° to 1100°C; Fig. 1B) (*15*). In oceanic lithosphere, the MLD deepens with seafloor age and systematically scales to 40 to 60% of the thermally defined LAB depth, with pervasive *V*_s_ drops of 3 to 15% over < 21 km (Fig. 1, C and D). Geophysical imaging further links the MLD to low-resistivity layers [e.g., the Slave craton (*28*)] and suggests enrichments in fluids or melt phases (*29*, *30*). Despite these compelling multidisciplinary constraints, a unified physical mechanism explaining the MLD’s genesis, persistence, and global ubiquity remains elusive.

Several hypotheses have been proposed to explain the origin of the MLD. These include (i) the presence of partial melt (*9*, *17*); (ii) the accumulation of volatile-bearing assemblages crystallized from partial melt and/or produced by metasomatism (*2*, *4*, *21*, *31*–*33*); (iii) change in seismic anisotropy (*34*); and (iv) elastically accommodated grain boundary sliding (EAGBS) (*15*, *35*). Each of these mechanisms, however, faces key limitations. Partial melt is unlikely to be stable at the relatively low temperatures characteristic of MLD depths (*1*). In the case of volatile-bearing phases, a central challenge is explaining how fluids or melts could be trapped and retained long enough to produce a seismically detectable signal. While EAGBS can reduce *V*_s_, the magnitude of this effect remains uncertain (*2*, *15*, *36*). Furthermore, the direct impact of grain size on *V*_s_ appears limited under expected MLD pressure-temperature conditions (*12*, *37*). Nevertheless, grain-size reduction could still play a key role by decreasing the permeability of the lithospheric mantle (*38*–*40*). This raises an intriguing possibility: Could the densification of the mantle lithosphere, driven by grain-size reduction, create permeability barriers that trap fluids or melts and further contribute to the formation of MLD?

In this study, we propose a framework to explain the global origin of MLD using thermomechanical models coupled with a self-consistent grain-size evolution process. Through numerical simulations at subduction zones, we found that MLD formation is mainly controlled by two dynamic processes: (i) deformation-induced grain-size reduction within the brittle-ductile transition governed by plate thermal structure and rheology, which markedly reduces permeability and creates an impermeable barrier; (ii) the accumulation and crystallization of fluids or melts beneath this barrier, resulting in the characteristic *V*_s_ reduction. This mechanism may be universally applicable across tectonic settings, including active margins, mature oceanic plates, and ancient cratons as grain-size reduction at the brittle-ductile transition is intrinsic to lithospheric deformation. By revealing how permeability barriers enable fluid retention, our framework directly links grain-scale processes to lithospheric-scale stratification and advances understanding of plate evolution by unifying rock microstructure, fluid migration, and mantle dynamics.

## RESULTS

### Numerical model setup and mantle dynamics

Our thermomechanical model solves the incompressible conservation equations for mass, momentum, and energy using a staggered finite-difference scheme with marker-in-cell techniques. The model domain spans 4000 km by 1400 km in the Eulerian frame (fig. S1). For the oceanic lithosphere, the thermal model incorporates the half-space cooling model and the plate cooling model [for plate age > 70 million years (Myr) assuming a constant plate thickness] (*41*, *42*). The continental lithosphere follows a linear thermal gradient. The asthenosphere is initially prescribed with a constant adiabatic thermal gradient of 0.5°C/km. Horizontal convergence at a rate of 5 cm/year is imposed inside the model on the subducting oceanic plate to mimic the far-field driving force. The model incorporates both brittle/plastic and viscous deformation mechanisms. Brittle/plastic deformation is governed by the strain-dependent Drucker-Prager criterion, whereas effective ductile viscosity is determined by harmonic averaging of dislocation and diffusion creep viscosities under a composite flow law (table S1) (*43*). The effective viscosity is defined as the lower value of the brittle/plastic and ductile viscosities, constrained within cutoff bounds of 10^18^ to 10^25^ Pa·s.

Grain-size evolution is modeled on the basis of a two-phase system comprising 60% olivine and 40% pyroxene (*44*). The grain-size evolution rate is composed of independent growth and reduction terms assisted by Zener pinning and depends on mechanical work, temperature, and pressure. Grain-size reduction occurs through dynamic recrystallization during dislocation creep, with the fraction of mechanical work partitioned into grain-size reduction following the formula of (*45*).

This reduction in grain size strongly affects mantle permeability and further modifies the pathways of rising fluids and melt phases (*38*–*40*). Using Darcy’s law, we calculate the permeability gradient within the mantle to identify potential barrier layers and fluid-enriched zones. To evaluate the impact of accumulated fluids on shear wave velocity, we use *Perple_X* (*39*, *46*) to calculate the seismic properties at both the MLD and the LAB. This integrated approach connects grain-size evolution and permeability variations to seismic properties, providing a comprehensive framework for investigating the mantle lithosphere properties.

### Numerical results on the MLD origin

**Fig. 2.**
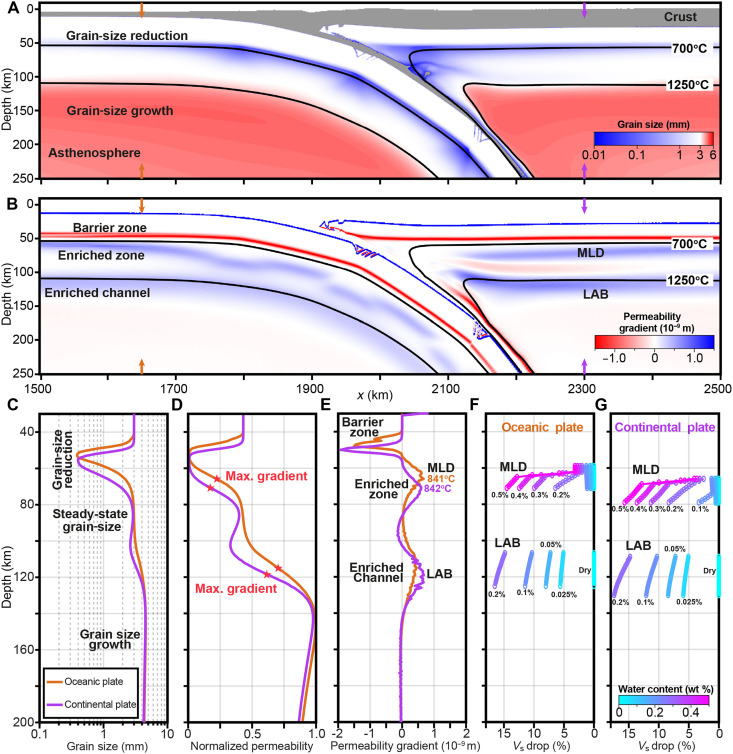
Numerical results at 19.7 Myr showing the formation of MLD in model *refer20o60*. The oceanic plate age is 60 Myr using a half-space cooling thermal model, and the continental plate thickness is 110 km. (**A**) Grain-size distribution. The gray color represents the crust where the grain-size effect is not considered, and the white color indicates the initial grain size. Grain-size reduction (blue) occurs at the brittle-ductile transition, while grain-size growth (red) is observed in the asthenosphere. (**B**) Permeability gradient calculated from grain-size field, highlighting the barrier layers and fluid-enriched zones. A low and negative permeability gradient layer at the brittle-ductile transition prevents vertical fluid flow, while two high and positive permeability gradient layers below the brittle-ductile transition and at the LAB indicate potential fluid enrichment zones. (**C** to **E**) Profiles of grain size, normalized permeability, and permeability gradient, with locations marked by arrows in (A) and (B). Max. gradient, indicated by the red star in (D), corresponds to the locations of the maximum permeability gradient shown in (E). (**F** and **G**) The effects of water content variation on *V*_s_ drop at MLD and LAB depths in both oceanic and continental lithosphere. The MLD and LAB are determined from the permeability gradient based on an average observed thickness of 15 km to 20 km (i.e., >3 × 10^−10^ m).

We perform two-dimensional (2D) thermomechanical modeling of oceanic-continental subduction to investigate the origin of MLD (Fig. 2). The simulations reveal significant grain-size variation across the lithosphere and asthenosphere (Fig. 2A). Grain-size reduction concentrates along the brittle-ductile transition zones in both the subducting oceanic plate and the overriding continental plate, where deviatoric stresses are high despite relatively low strain rates (figs. S2 and S3). The resulting mechanical work rate is substantial (Eq. 13) and drives grain-size reduction within the dislocation creep regime (fig. S4), mediated by dynamic recrystallization (*45*). As grain size decreases, deformation progressively shifts toward diffusion creep dominance, which reduces the mechanical work available for further recrystallization-driven reduction and drives grain size toward a quasi-steady state. This rheological feedback promotes the development of thin, low-permeability zones in both plates. In the subducting plate, this process also contributes to bending-induced slab segmentation (*43*). In contrast, the asthenosphere, with its higher temperatures and dominant diffusion creep, exhibits pervasive grain growth and maintains larger grain sizes.

Using Darcy’s law, we compute the vertical permeability gradient from the modeled grain-size field. This analysis identify one permeability barrier at the brittle-ductile transition, where the grain size decreases sharply (Fig. 2B, red color, characterized by a high negative permeability gradient), and two fluid-enriched layers, where the grain size varies from small to large (Fig. 2B, blue color, marked by a high positive permeability gradient). The barrier zone forms with the minimum grain size (<0.5 mm, over 10 km to 20 km; Fig. 2C) and very low permeability (Fig. 2D). Below this layer, grain size remains close to its initial value (white color) in a quasi-steady state within the rigid undeformed lithosphere, producing a potential enrichment layer characterized by a rapid permeability variation (Fig. 2D). This enrichment layer with peak positive permeability gradient locates at a depth of around 66 km in the oceanic lithosphere and 71 km in the continental lithosphere (Fig. 2E), with temperatures around 840°C for both. These values are generally consistent with geophysical observations of the MLD (Fig. 1). On the other hand, temperature-driven grain growth within the hotter asthenosphere produces a strong permeability contrast and an additional potential enrichment zone at the LAB (Fig. 2, A and B).

To assess seismic consequences, we calculate *V*_s_ drops resulting from fluid accumulation in these enrichment layers using thermodynamic modeling (*39*, *46*). For a representative permeability gradient exceeding 3 × 10^−10^ m (estimated for a 15- to 20-km-thick enrichment zone), our simulations show that the *V*_s_ drop is highly sensitive to water content (fig. S5). Even 0.2 wt % water can cause *V*_s_ reductions of 5% in the oceanic lithosphere and 8% in the continental lithosphere at MLD conditions (2.5 GPa, 800°C; Fig. 2, F and G). Only 0.1 wt % water is required to achieve a 10% *V*_s_ drop near the LAB in both oceanic and continental settings. We also calculate the direct effect of grain size on *V*_s_ using the Very Broadband Rheology Calculator (VBRc) (*37*), which accounts for frequency-dependent viscoelastic properties. While grain-size reduction significantly reduces *V*_s_ at LAB-like temperatures (>1300°C) (*47*), it has a negligible impact at the lower temperatures characteristic of the MLD (600° to 1100°C; fig. S6).

These results indicate that deformation-driven grain-size reduction at the brittle-ductile transition forms a low-permeability barrier that traps fluids, creating a fluid-enriched layer responsible for the MLD. In contrast, grain-size growth within the asthenosphere promotes the development of an additional fluid- or melt-enriched zone associated with the LAB. Both features correspond to observed *V*_s_ reductions and align well with global geophysical observations (Fig. 1). In addition, a sensitivity test with a spatially heterogeneous (randomly perturbed) initial grain-size field yields the same first-order permeability contrasts and potential enrichment layers (fig. S7), demonstrating the robustness of the proposed mechanism.

### Temperature and rheology controls on MLD variability

**Fig. 3.**
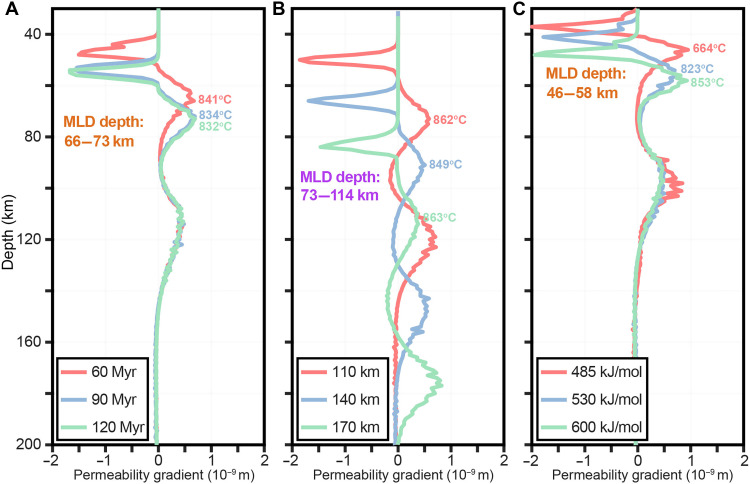
Sensitivity analyses of the depth and temperature distribution of peak permeability gradient. (**A**) Variation in oceanic MLD depth as a function of oceanic plate age, showing results for a 60-Myr-old oceanic plate (half-space cooling model) and 90- and 120-Myr-old oceanic plates (plate cooling model with a plate thickness of 100 km). (**B**) Dependence of continental MLD depth on continental plate thickness. (**C**) Influence of activation energy for dislocation creep on the oceanic MLD. Profile locations are shown in Fig. 2.

The MLD exhibits substantial variability in depth and temperature in nature (Fig. 1). Our systematic thermomechanical modeling across diverse thermal and rheological regimes (table S2) demonstrates that MLD depth is primarily governed by lithospheric thermal structure, while deformation mechanisms fine-tune its depth. In hot lithospheres, such as young oceanic plates or thin continental plates, the brittle-ductile transition occurs at shallow depths, forming a shallow low-permeability barrier and a correspondingly shallow MLD. For example, in a 60-Myr-old oceanic plate, the MLD lies at 66 km; in a 110-km-thick continental plate, it appears at 73 km. In contrast, cold lithospheres, such as old oceanic plates or thick cratonic lithosphere, have deeper brittle-ductile transitions and therefore deeper MLD, up to 70 km to 80 km in ancient oceanic plates and 115 km in cratonic interiors (Fig. 3, A and B).

Given the ongoing debate over oceanic lithospheric thickness, we assess the influence of half-space cooling and plate cooling models on MLD depth using thermal profiles constrained by geophysical observations (*4*, *41*). Both thermal models exhibit consistent thermal configurations for oceanic plates younger than 70 Myr (*42*). Under the plate cooling model, for a 90-Myr-old oceanic plate, increasing the plate thickness from 95 km to 120 km leads to an MLD depth increase from 67 km to 76 km. Similarly, as the oceanic plate age increases to 120 Myr, the MLD depth rises slightly from 72 km to 81 km (table S2). The half-space cooling model predicts slightly deeper MLD at older ages, but, overall, the MLD consistently clusters around 70 km. By contrast, continental MLD depth varies widely, increasing with lithospheric thickness (Fig. 3B). Despite these differences in depth, MLD temperatures are remarkably consistent, 830° to 850°C in the oceanic lithosphere (Fig. 3A) and 840° to 870°C in the continental lithosphere (Fig. 3B), well below solidus (*1*). The thickness of the MLD layer is also comparable across settings, typically 10 km to 20 km, consistent with seismic reflections in the oceanic lithosphere (*18*, *48*) and low-resistivity zones in cratons (*28*).

Beyond thermal structure, rheological mechanisms also influence the depth and thermal regime of the MLD. The activation energy in dislocation creep directly influences dislocation viscosity, shifting its intersection with brittle strength (figs. S2, S4, and S8), which defines the depth of the brittle-ductile transition. A lower activation energy (e.g., 485 kJ/mol) reduces dislocation viscosity, resulting in a shallower transition and a lower temperature at that depth (Fig. 3). In contrast, as activation energy increases to 530 to 600 kJ/mol, dislocation viscosity increases, pushing the transition deeper into hotter regions. Steep permeability gradients emerge immediately below this transition (Fig. 3C), defining fluid-enriched layers that produce the MLD. Within the explored rheological parameter space for the oceanic lithosphere, MLD depths range from 46 km to 58 km, with corresponding temperatures of 664° to 853°C. This variability directly reflects how rheology fine-tunes both the position and thermal state of the MLD.

## DISCUSSION

**Fig. 4.**
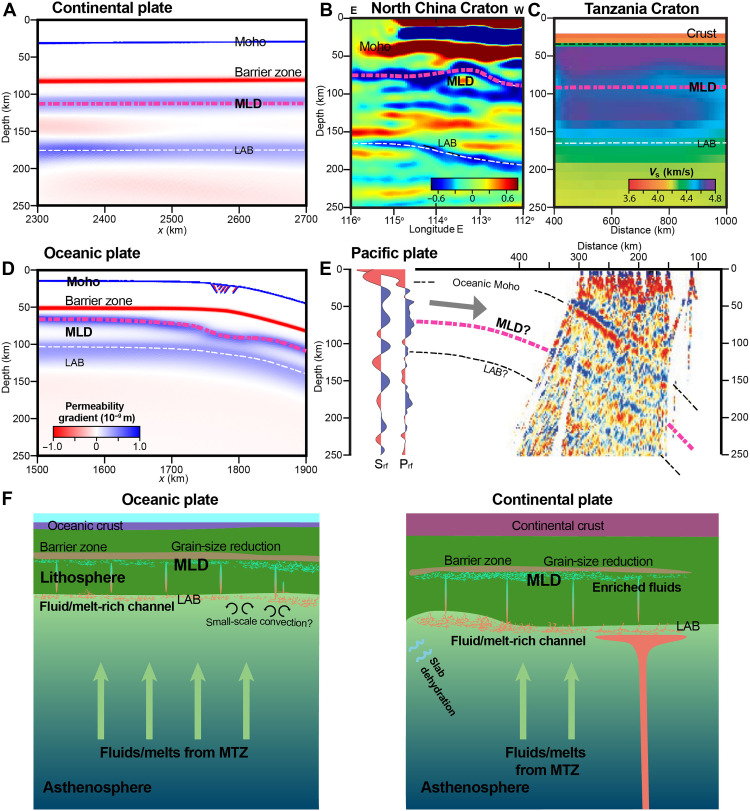
Natural evidence and schematic diagram of MLD formation. (**A** to **C**) Comparison between the model result (left: model *refer20cth180*) and two natural examples of continental lithospheric layering: the S receiver function (RF) image of the North China Craton (middle) (*24*), and the S-wave velocity structure image of the Tanzania Craton (right) (*51*). (**D** and **E**) Comparison between the model result (left: model *refer20o90pl95*, using a plate cooling model with a plate thickness of 95 km) and a natural example of the subducting Pacific Plate with an age of ~129 Myr (right), including vertical profiles of S-RF and P-RF amplitudes and the P-RF image [modified with permission from Kawakatsu *et al.* (*52*)]. Coherent velocity discontinuities inferred from S-to-P and P-to-S waves are indicated in (B) and (E), respectively. Panels (A) and (D) share the same color scale. (**F**) Schematic diagram illustrating formation mechanisms of MLD in oceanic (left) and continental (right) lithosphere.

Based on the numerical results, we propose that the reduced mantle permeability, caused by grain-size reduction at the brittle-ductile transition, plays an important role in trapping the upwelling fluids and volatiles from the subjacent mantle (*49*, *50*). This process creates a fluid enrichment layer, resulting in a sharp *V*_s_ drop within the lithosphere. Grain-size reduction at the brittle-ductile transition is a fundamental aspect of lithospheric deformation (*43*) driven by large-scale plate motions. By focusing on this mechanism, our approach isolates the grain-size-rheology-permeability feedbacks relevant to MLD formation without relying on specific plate-velocity histories while remaining valid with stress levels expected in the natural tectonic systems with significant far-field stresses present in both oceanic and continental plates. The proposed mechanism is therefore expected to operate equivalently across diverse tectonic settings, which may offer a self-consistent, unifying explanation for the MLD in both oceanic and continental lithosphere (Fig. 4). In the ancient cratons, clear MLD seismic velocity discontinuities have been documented (Fig. 4, B and C) in regions such as the North China Craton and Tanzania Craton (*24*, *51*). These observations align closely with our model results for a 170-km-thick continental lithosphere (Fig. 4A). Similarly, for the oceanic lithosphere, receiver function analyses beneath the Pacific and Philippine Sea plates reveal several sharp seismic velocity contrasts (*52*). For the old Pacific plate (129 Myr), the shallow discontinuity within the lithosphere occurs at a depth of 80 km to 82 km (Fig. 4E), which aligns well with our model predictions in a 120-Myr-old oceanic plate using the plate cooling model (with a plate thickness of 95 km), where the MLD depth is around 72 km to 78 km (Fig. 4D). Overall, our models show a strong correspondence with geophysical observations, validating their applicability to both oceanic and continental lithospheres (Fig. 4F). They further elucidate the intricate interplay of melt dynamics, fluid migration, and accumulation processes that control the formation and characteristics of MLD.

The potential sources of the accumulating fluids in this mechanism include upwelling fluid/melt from the mantle transition zone or mantle plumes (*53*, *54*), asthenospheric shear flow (*55*), and slab-derived fluids from subduction (*56*) (Fig. 4F). These fluids (notably, H_2_O and CO_2_) preferentially partition into melts, enriching small melt fractions (~0.1%) even at low bulk concentrations (*57*, *58*). Such volatile-rich melts can accumulate beneath the lithosphere, forming a thin (10 km to 20 km) channel at its base, as imaged by ultradeep seismic reflections (*59*) and evidenced by low-velocity zones (*59*–*63*) and high-conductivity layers (*64*). This channel likely arises from the accumulation of deep-mantle melts or from lateral melt/fluid migration along the inclined lithosphere base, potentially facilitated by small-scale convection. Beneath stable cratons, fluid upwelling from the mantle transition zone combines with slab dehydration or mantle plume activity (*65*) to enhance fluid accumulation (Fig. 4F).

The MLD is observed across a wide range of tectonic environments (Fig. 1), most prominently in active margins, subduction zones, and also within cratonic interiors that experienced significant tectonic activity either during ancient craton assembly or in more recent reworking events (*66*). Beyond subduction, orogenic collision and oceanic extension similarly promote the reduction of grain size at the brittle-ductile transition through mechanical deformation (*43*, *67*). In contrast, mid-ocean ridges are characterized by a thin, melt-rich lithosphere dominated by melt migration (*68*, *69*), rather than by grain-size feedbacks in a cooling, mature lithosphere, making MLD formation unlikely at ridge axes. In addition to deformation-driven permeability contrasts, stress-controlled mechanisms, such as the variation in the mean stress gradient below the brittle-ductile transition, may further enhance fluid ponding (*70*, *71*). Together, these processes imply that the lateral extent and persistence of grain-size reduction and associated low-permeability layers scale with the intensity and longevity of tectonic forcing.

Once established, reduced grain sizes exhibit exceptionally slow healing kinetics (*72*), requiring hundreds of millions of years to recover even at high temperatures (>900°C) in the absence of mechanical deformation (fig. S9). At lower temperatures (<900°C), grain-size reduction persists within the brittle-ductile transition zone (fig. S9), and, under tectonic deformation (higher differential stress), the reduction is strongly accelerated, allowing grain size to decrease rapidly (*73*). As grain size shrinks, the reduction rate diminishes, and the system approaches a quasi-steady state. This explains pervasive MLD detection at 70- to 100-km depths (*27*, *74*), where tectonic deformation events established permanent permeability barriers that trap fluids.

The MLD regulates mantle-to-lithosphere volatiles transfer by permeability contrasts that trap ascending fluids/melts. Fluid accumulation beneath the barrier, along grain boundaries and/or within volatile-bearing minerals, may locally weaken the lithosphere. However, despite local fluid accumulation, viscosity at the MLD depths can still remain sufficiently high (*16*) to inhibit significant rheological decoupling across the interface under ambient conditions, consistent with our results in figs. S2 and S3. Crucially, this mechanical stability can be weakened by abrupt thermal perturbations associated with asthenospheric upwelling, whether driven by subduction dynamics or mantle plumes (*62*, *75*, *76*). Such thermal processes rapidly lower viscosity within the MLD layer and facilitate lithospheric delamination and thinning, positioning the MLD as both a stabilizing layer and a potential failure zone. Its dual behavior is governed by the interplay among fluid/melt accumulation, grain-size reduction, and thermal perturbations, allowing the MLD to act as a dynamic modulator of lithospheric evolution.

In contrast, the lithosphere and asthenosphere become decoupled at the LAB, where the weak and hot mantle promotes grain growth and facilitates the formation of a melt-rich channel, as predicted by our models. However, the detailed pathways and mechanisms by which melt migrates from the deep mantle toward the LAB and MLD remain poorly understood and warrant further investigation using high-resolution two-phase flow (i.e., fluid-solid) models (*49*) coupled with grain-size evolution. Furthermore, mantle grain-size variability, particularly at plate boundaries, and its mechanical effects likely play an active role in initiating key tectonic processes, including rifting, subduction initiation, and volcanism, by acting as a strain localization zone (*77*).

This framework establishes grain-scale processes as fundamental controls on lithospheric stratification, reconciling global MLD observations through thermo-rheological feedbacks. A comprehensive understanding of lithospheric stratification is thus essential for deciphering the complex interactions between melt/fluid cycling, mantle-lithosphere-crust interactions, and surface deformation throughout Earth’s history. Advancing our knowledge of lithospheric stratification not only addresses fundamental geodynamic questions but also opens avenues for interpreting the tectonic and thermal evolution of planetary interiors across the solar system.

## MATERIALS AND METHODS

The thermomechanical 2D numerical code I2VIS is used in this study (*43*, *78*). The specific version of the code follows (*43*). The momentum, mass, and energy conservation equations are solved in an Eulerian frame, and physical properties are transported by Lagrangian markers. A visco-plastic rheology is adopted, in which the brittle/plastic (η_plastic_) and viscous (η_ductile_) behavior of the lithospheric plate (table S1) is defined by experimentally determined flow laws (*79*).

### Brittle/plastic rheology

For the brittle/plastic deformation, its rheology is controlled by fracture-related strain weakening. The rheology follows a strain-dependent Drucker-Prager yield criterion

$$\eta_{\text{plastic}}=\frac{\sigma_{\text{yield}}}{2{\dot{\varepsilon }}_{\text{II}}}$$ (1)

$$\sigma_{\text{yield}}=C+\mu P$$ (2)

where η_plastic_ is the effective plastic viscosity, σ_yield_ is the scalar yield stress, and ${\dot{\varepsilon }}_{\text{II}}$ is the second invariant of the strain rate tensor. *P* is the pressure. *C* is the cohesion (0.3 MPa, rock strength at *P* = 0), and μ is the effective internal friction coefficient, which depends on the accumulated plastic strain $\gamma =\int \sqrt{\frac{1}{2}{\left({\dot{\varepsilon }}_{\mathrm{ij}\left(\text{plastic}\right)}\right)}^{2}}\mathrm{dt}$ [time integrating the second invariant of the plastic strain rate tensor ${\dot{\varepsilon }}_{\mathrm{ij}\left(\text{plastic}\right)}$], defined by

$$\mu \left(\gamma \right)=\left\{ \begin{array}{ll} \mu_{0}, & \gamma \leq \gamma_{0}\\ \mu_{0}+\frac{\gamma -\gamma_{0}}{\gamma_{1}-\gamma_{0}}\left(\mu_{1}-\mu_{0}\right), & \gamma_{0}<\gamma \leq \gamma_{1}\\ \;\mu_{1}, & \gamma >\gamma_{1} \end{array}\right.$$ (3)

where μ_0_ and μ_1_ are the initial and final friction coefficients, respectively, with μ_0_ ≥ μ_1_. The difference Δμ = μ_0_ − μ_1_ characterizes the intensity of strain weakening.

### Ductile rheology

Ductile deformation is described using a composite rheology, where the effective viscosity η_ductile_ is the harmonic average of diffusion creep viscosity (η_df_) and dislocation creep viscosity (η_ds_)

$$\frac{1}{\eta_{\text{ductile}}}=\frac{1}{\eta_{\text{df}}}+\frac{1}{\eta_{\text{ds}}}$$ (4)

For crustal rocks, grain-size evolution is not considered. η_df_ and η_ds_ are computed using the simplified flow law (*43*)

$$\eta_{\text{df}}=\frac{A_{D}}{2\sigma_{\text{cr}}^{n-1}}\text{exp}\left(\frac{E+\mathrm{PV}}{\mathrm{RT}}\right)$$ (5)

$$\eta_{\text{ds}}=\frac{1}{2}A_{D}^{1/n}\text{exp}\left(\frac{E+\mathrm{PV}}{\mathrm{nRT}}\right){\dot{\varepsilon }}_{\text{II}}^{\left(1/n\right)-1}$$ (6)

where *R* is the gas constant, *T* is temperature, and *n* is the experimentally determined stress exponent for the dislocation creep. *A_D_*, *E*, and *V* are, respectively, experimentally determined prefactor, activation energy, and activation volume, which are assumed to be the same for both diffusion and dislocation creep. The transition between diffusion and dislocation creep is controlled by the critical stress σ_cr_ (*42*).

For mantle rocks, the ductile creep model considers grain size, which is computed through Zener pinning in a two-phase medium. The grain size is implemented in the diffusion creep as follows

$$\eta_{\text{df}}=\frac{1}{2A_{\text{df}}}{\left(\frac{\pi r}{2}\right)}^{m}\text{exp}\left(\frac{E_{\text{df}}+{\mathrm{PV}}_{\text{df}}}{\mathrm{RT}}\right)$$ (7)

where *E*_df_ and *V*_df_ are the activation energy and volume, *A*_df_ is the prefactor, and *m* is the grain-size exponent. The grain interface roughness *r* relates to the average grain size *d* by $d\approx \frac{\pi r}{2}$ (*72*, *80*, *81*).

The dislocation creep viscosity is defined through the following equations

$$\eta_{\text{ds}}=\frac{1}{2A_{\text{ds}}}\sigma^{1-n}\text{exp}\left(\frac{E_{\text{ds}}+{\mathrm{PV}}_{\text{ds}}}{\mathrm{RT}}\right)$$ (8)

$$\sigma =2\eta_{\text{ductile}}{\dot{\varepsilon }}_{\text{II}}$$ (9)

$$\sigma \leq \sigma_{\text{yield}}$$ (10)

where *E*_ds_, *V*_ds_, and *A*_ds_ are the activation energy, volume, and prefactor for dislocation creep. *n* is the dislocation creep exponent. The ductile stress σ is limited by the yield stress defined in Eq. 2. Equations 8 to 10 are solved iteratively.

The effective viscosity (η_eff_) is defined as the minimum of the brittle/plastic and ductile viscosities, constrained within cutoff bounds of 10^18^ to 10^25^ Pa·s.

$$\eta_{\text{eff}}=\text{min}\left(\eta_{\text{plastic}},\;\eta_{\text{ductile}}\right)$$ (11)

The dominant rheology in the ductile part is calculated using viscosity ratios

$$\left\{\begin{array}{ll} \frac{\eta_{\text{df}}}{\eta_{\text{ds}}}\leq 0.1, & \text{diffusion creep dominates}\\ 0.1\;<\frac{\eta_{\text{df}}}{\eta_{\text{ds}}}\leq 1.0, & \text{Transition}\;I\\ 1.0<\frac{\eta_{\text{df}}}{\eta_{\text{ds}}}\leq 10.0, & \text{Transition}\;\text{II}\\ \frac{\eta_{\text{df}}}{\eta_{\text{ds}}}>10.0, & \text{dislocation creep dominates} \end{array}\right.$$ (12)

In Transition I, diffusion creep dominates, but with some dislocation creep. In Transition II, dislocation creep dominates, but with some diffusion creep. An example of the relationship between brittle/plastic deformation, diffusion creep, dislocation creep, ductile viscosity, and the final effective viscosity is presented in figs. S4 and S8. The rheological transition from brittle to ductile deformation is smooth and depends on the temperature and strain rate distribution.

### Grain-size evolution

In mantle material, the interplay between diffusion and dislocation creep is therefore related to a grain-size evolution equation that depends on the mechanical work, temperature, and pressure. The grain-size evolution model relies on several assumptions:

1) Mantle peridotite is assumed to be composed of two well-mixed phases: olivine and pyroxene, with a fixed volume fraction of 60 and 40%, respectively. For simplicity, these phases are considered to have the same density and rheology.

2) The relative motion between the two phases is neglected; their velocities are identical.

3) It is assumed that the grain-size distribution is self-similar and log-normal (*45*). Therefore, it always retains the same shape, and its mean variance and amplitude are fully characterized by a unique mean grain size.

The system is in a state known as pinned state limit (*72*, *81*), wherein the grain-size evolution is limited by the pinning of phases by each other (i.e., Zener pinning). In these conditions, the grain size is controlled by the roughness *r* of the interface between the two phases. The mean grain size is given by $d\approx \frac{\pi r}{2}$. The roughness evolution is described by the following equations (*72*, *80*, *81*)

$$\frac{\mathrm{dr}}{\mathrm{dt}}=\frac{\eta G_{I}}{{\mathrm{qr}}^{q-1}}-\frac{f_{I}r^{2}}{\gamma_{I}\eta }\Psi$$ (13)

$$G_{I}=\frac{G_{g}}{G_{\text{fac}}}\frac{q}{p}\;r^{q-p}$$ (14)

$$G_{g}=A_{g}\text{exp}\left(-\frac{E_{g}+{\mathrm{PV}}_{g}}{\mathrm{RT}}\right)$$ (15)

$$f_{I}=f_{0}\text{exp}\left[-2{\left(\frac{T}{1000}\right)}^{2.9}+2\right]$$ (16)

where *G*_I_ is the interface coarsening rate, *G*_g_ is the grain growth rate, and *G*_fac_ = 100 is a dimensionless grain growth factor. The parameter *q* = 4 is the roughness coarsening exponent, and *P* = 2 is the grain-size coarsening exponent. The surface tension is given by γ_I_ = 1.0 Pa·m. The preexponential factor for grain growth is *A*_g_ = 2 × 10^4–6*p*^, and the activation energy is *E*_g_ = 3 × 10^5^ J/mol. The activation volume is *V*_g_ = *V*_df_, associated with the grain growth process. The parameter *f*_I_ represents the fraction of mechanical work Ψ converted to interface damage, resulting in grain-size reduction. At *T* = 1000 K, *f*_0_ = 0.001. The interface area density is η = 3ϕ_ol_ϕ_px_, where ϕ_ol_ = 0.6 and ϕ_px_ = 0.4 are the volume fractions of olivine and pyroxene, respectively, in the mantle.

### Permeability in the mantle

Based on Darcy’s law, mantle permeability (κ) can be expressed as a function of porosity and grain size within the solid aggregate (*38*): $\kappa =\frac{d^{2}\phi^{n}}{C}$, where ϕ is the melt- or fluid-filled porosity, and *C* and *n* are (semi)theoretically derived constants related to the geometry of the pore network. Here, we define the dimensionless prefactor κ_0_ = ϕ*^n^*/*C* and focus on the effect of grain size *d* on mantle permeability. To quantify how permeability varies spatially, we compute the permeability gradient, which is used to identify potential barriers and enriched zones

$$\frac{\partial }{\partial y}\left(\frac{\kappa }{\kappa_{0}}\right)=2d\frac{\partial d}{\partial y}$$ (17)

### Thermodynamic simulations

Equilibrium mineral assemblages as a function of pressure and temperature were computed in the K_2_O-Na_2_O-SiO_2_-Al_2_O_3_-CaO-MgO-FeO-TiO_2_-Cr_2_O_3_-O_2_-H_2_O system using *Perple_X* (*46*) and the *hp633ver.dat* thermodynamic database. Mineral properties and solution models from the dataset of Holland *et al*. (*82*) were selected to incorporate the various end members of minerals typically found in mantle lithologies. Based on the fluid-enriched zones identified near the MLD and LAB, we assess how variations in water content affect shear wave velocities in these regions.

## Supplementary Materials

### This PDF file includes:

Figs. S1 to S9 Tables S1 and S2 References
