# Supplementary material for: Deciphering grain-size reduction as a driver of mid-lithosphere discontinuity formation
Source: Sci Adv. 2026 Apr 24;12(17):eaed4229. doi: 10.1126/sciadv.aed4229 (PMC13108559; doi:10.1126/sciadv.aed4229)
Supplement: Supplementary file 1 — Figs. S1 to S9 Tables S1 and S2 References [file sciadv.aed4229_sm.pdf]

Science Advances
AAAS

Supplementary Materials for

Deciphering grain-size reduction as a driver of mid-lithosphere discontinuity formation

Mingqi Liu *et al.*

Corresponding author: Mingqi Liu, liumq12@outlook.com; Zhong-Hai Li, li.zhonghai@ucas.ac.cn

Sci. Adv. **12**, eaed4229 (2026)
DOI: 10.1126/sciadv.aed4229

This PDF file includes:

Figs. S1 to S9
Tables S1 and S2
References

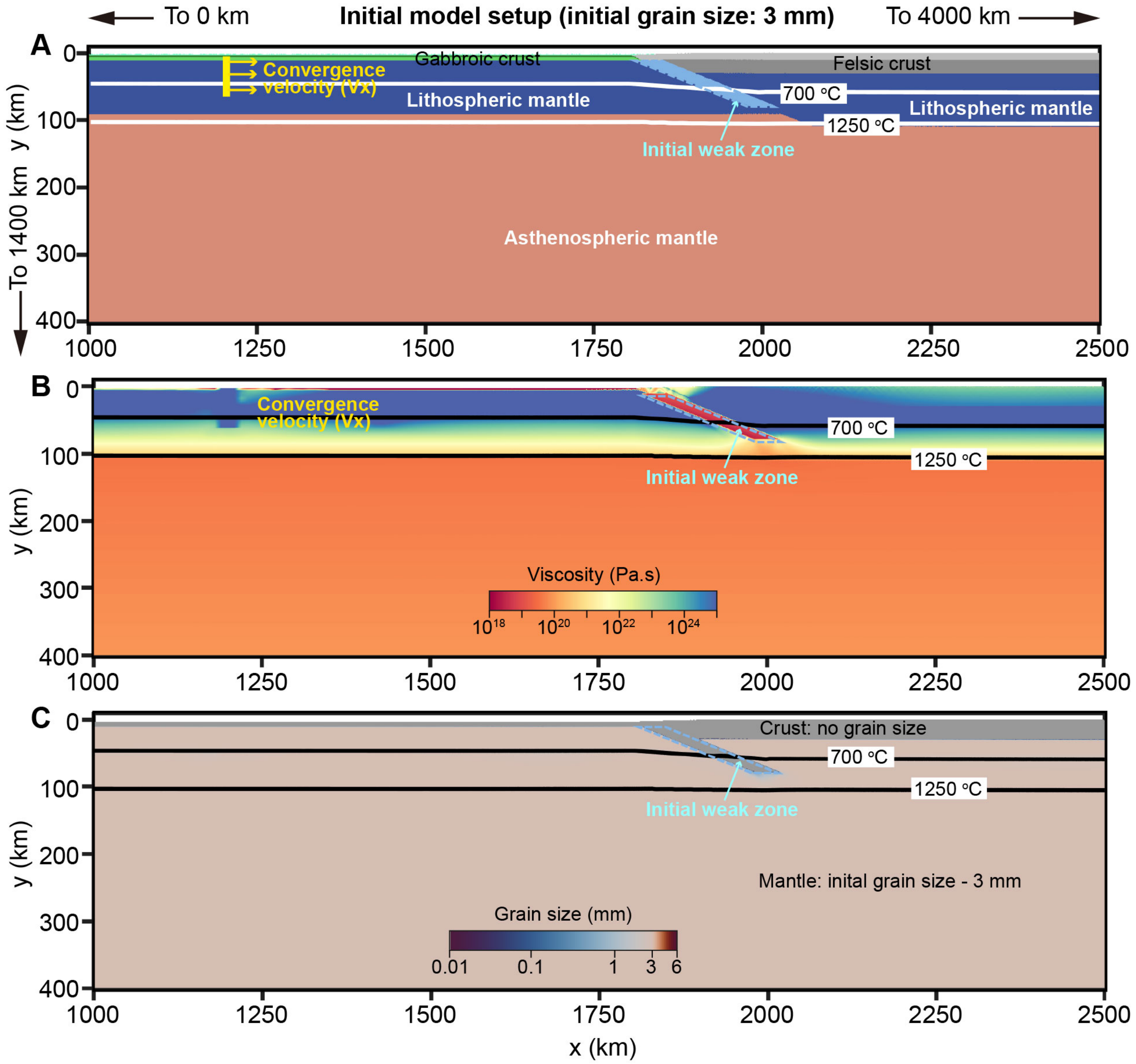

Figure S1: **Initial setup of the oceanic-continental subduction model.** (**A**) Composition field configuration and imposed initial conditions. A weak zone is prescribed at the passive margin, and a constant internal prescribed velocity is applied at x = 1200 km from the crustal surface to a depth of 70 km within the oceanic plate. Sticky air (i.e., air and water) is implemented to simulate the topography. The model domain spans 4000 km horizontally and 1400 km vertically. (**B**) Initial viscosity distribution. The weak zone creates a weak channel that controls the initial subduction style. Very high viscosity is implemented in the imposed internal velocity block. (**C**) Initial grain size distribution. Grain size is not considered in the crust and weak zone, marked by a grey color. The initial grain size in the lithosphere and asthenosphere is uniformly set to 3 mm. To clearly display the initial grain size, a colormap different from that used in the other figures is adopted.

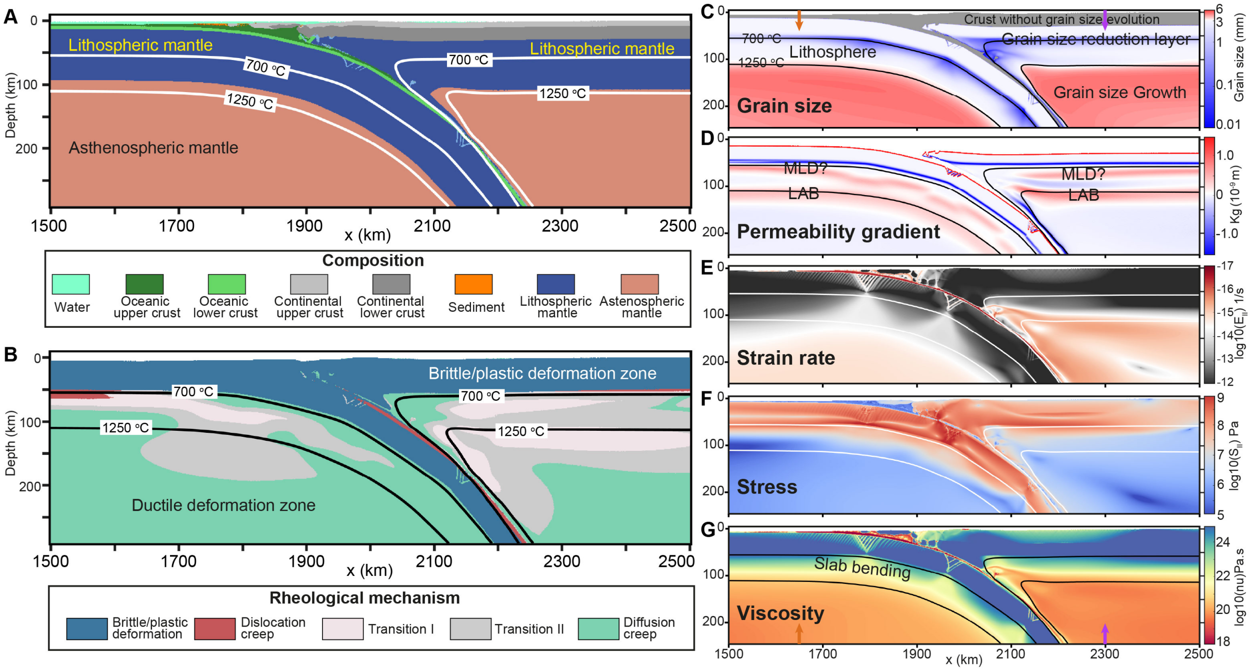

Figure S2: **Numerical results at 19.7 Myr for model `refer20o60`.** (**A**) Composition. White lines show the 700 and 1250 °C isotherms. (**B**) Rheological mechanism. The local effective viscosity is determined by the minimum value between the brittle/plastic and ductile viscosity, further constrained by cut-off values (*67*). (**C**) Grain size distribution. The grey color marks the crust whose rheological properties are not dependent on the grain size. (**D**) Permeability gradient. (**E**) Second invariant of strain rate. (**F**) Second invariant of stress. (**G**) Viscosity bounded between 10^{18}–10^{25} Pa·s. Arrows in (**C**) and (**G**) indicate the profile locations shown in Fig. S3.

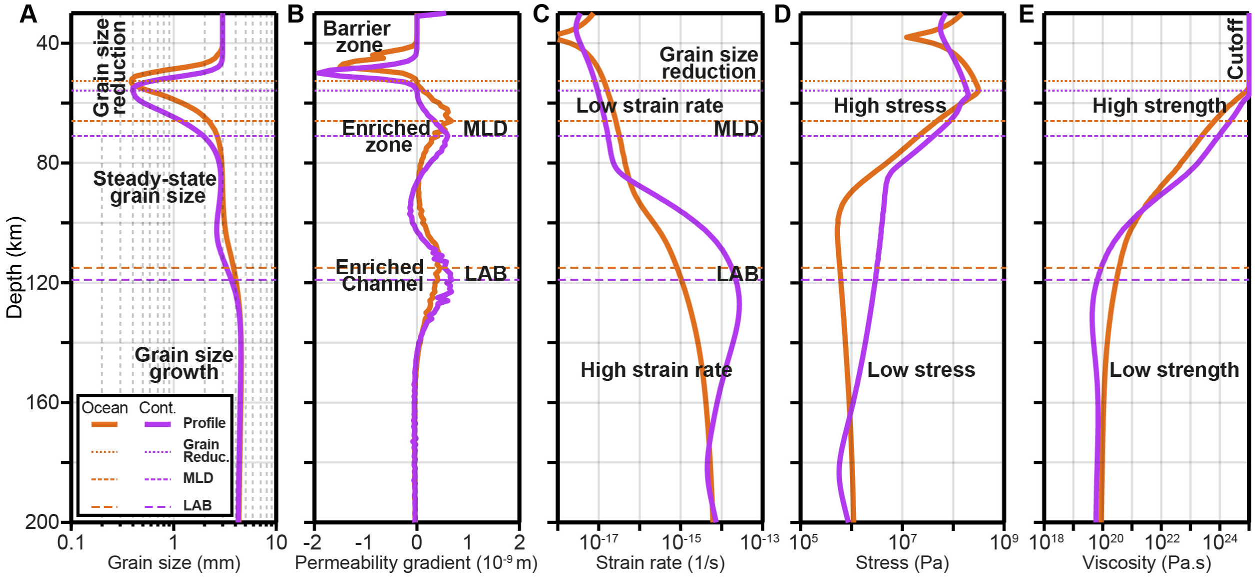

Figure S3: **Vertical profiles within both oceanic and continental plates.** Profiles correspond to locations indicated in Fig. S2 (**C–G**): $x = 1650$ km within the oceanic plate and $x = 2300$ km within the continental plate. (**A**) Grain size. (**B**) Permeability gradient. (**C**) Second invariant of strain rate. (**D**) Second invariant of stress. (**E**) Viscosity. Cont., continent. Grain Reduc., grain size reduction. High stress at the brittle–ductile transition (Fig. S4) drives significant grain size reduction even where strain rates are low, whereas high temperatures in the asthenosphere promote grain growth.

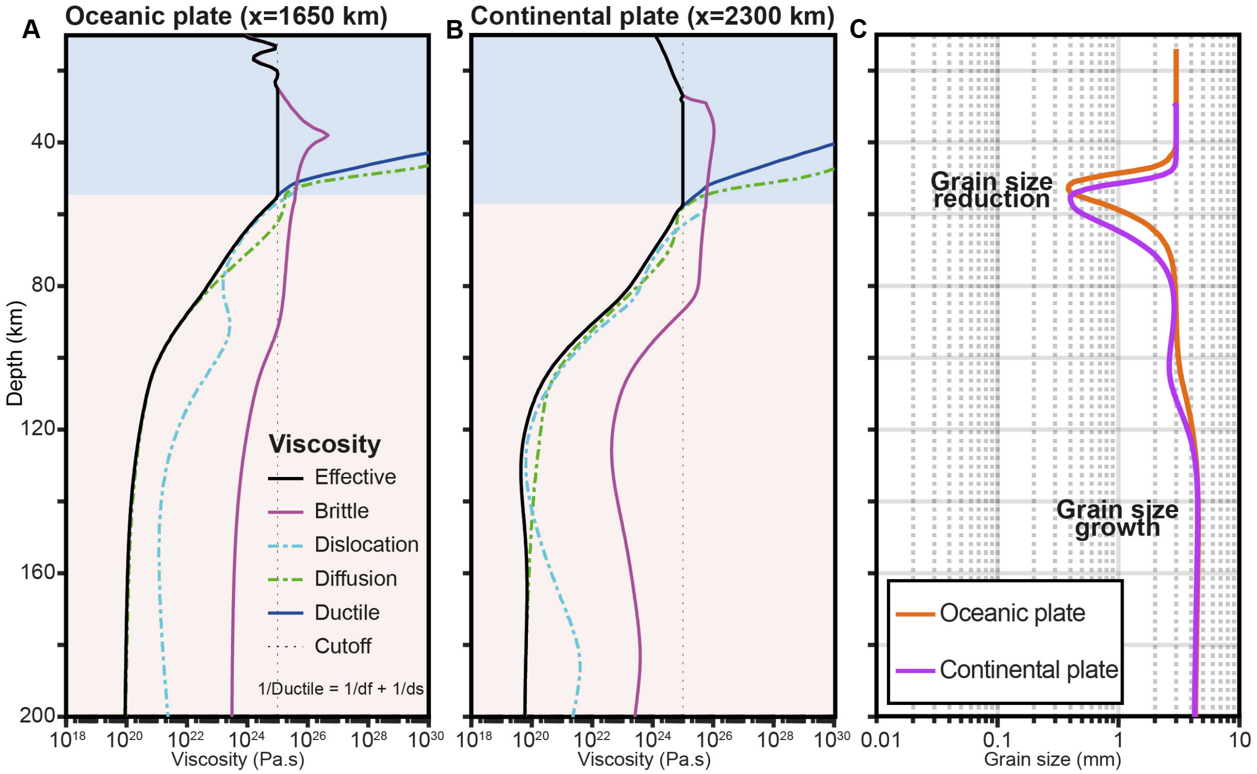

Figure S4: **Analysis of rheological mechanisms and grain size as shown in Fig. S2.** (**A**) Dominant rheological mechanism along the $x = 1650$ km profile in the oceanic domain. (**B**) Dominant rheological mechanism along the $x = 2300$ km profile in the continental domain. (**C**) Grain size profiles for both the oceanic and continental domains.

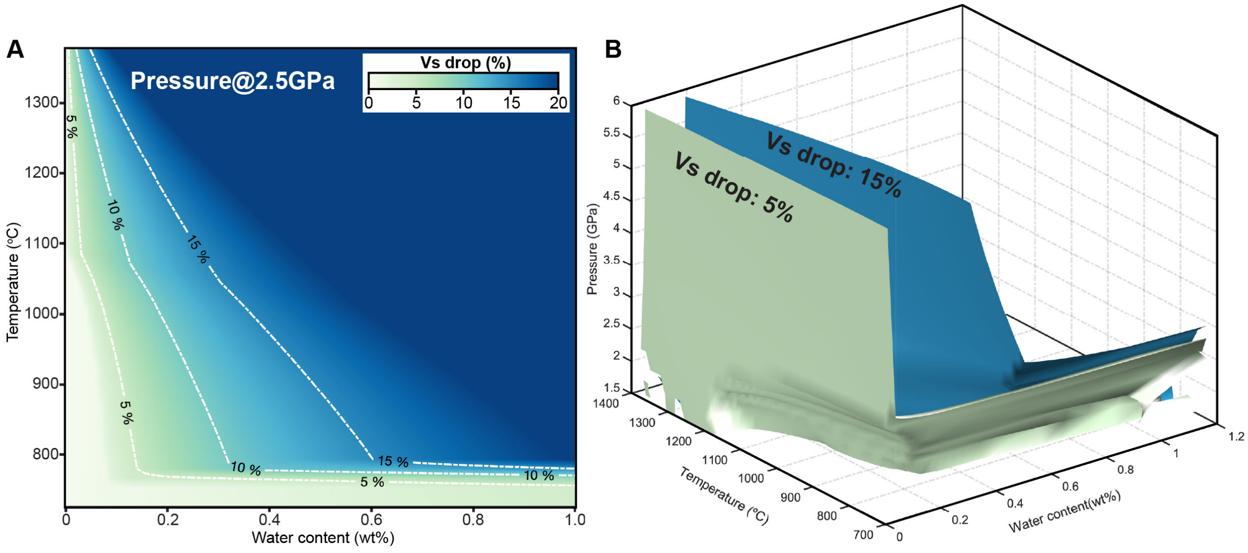

Figure S5: **Thermodynamic simulations using `Perple_X`.** (**A**) Shear wave velocity drop as a function of water content and temperature under a constant pressure of 2.5 GPa. (**B**) Isosurfaces for 5% and 15% V_s drop as a function of pressure, temperature, and water content.

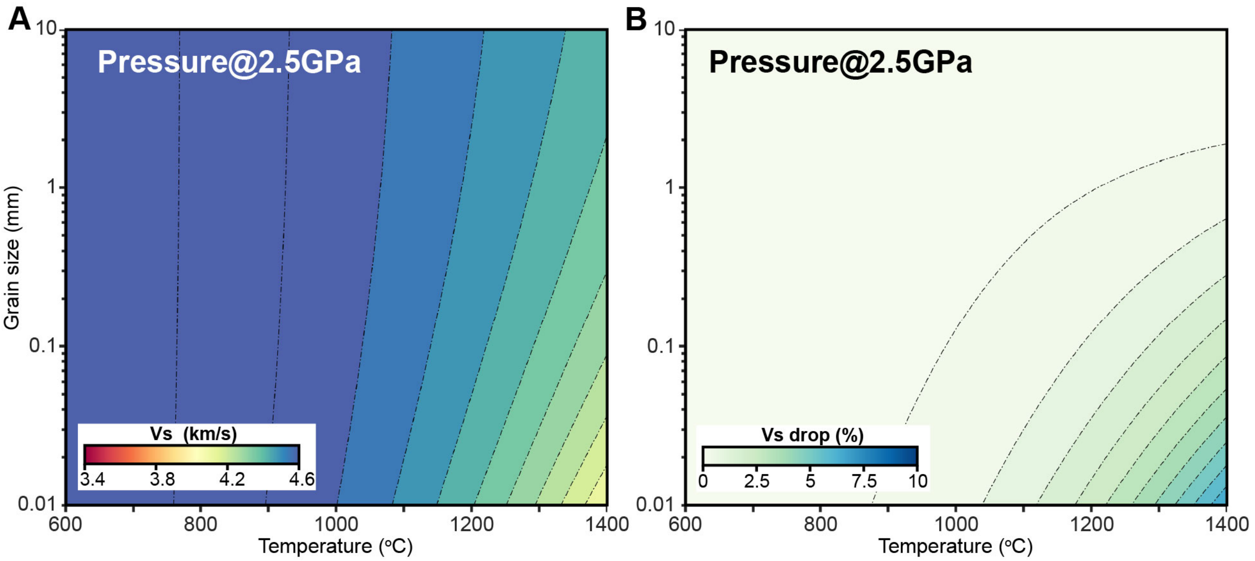

Figure S6: **Shear wave velocity as a function of temperature and grain size.** Calculations were performed using the Very Broadband Rheology Calculator (VBRc) (*37*) at a constant pressure of 2.5 GPa. (**A**) Shear wave velocity. (**B**) Shear wave velocity drop.

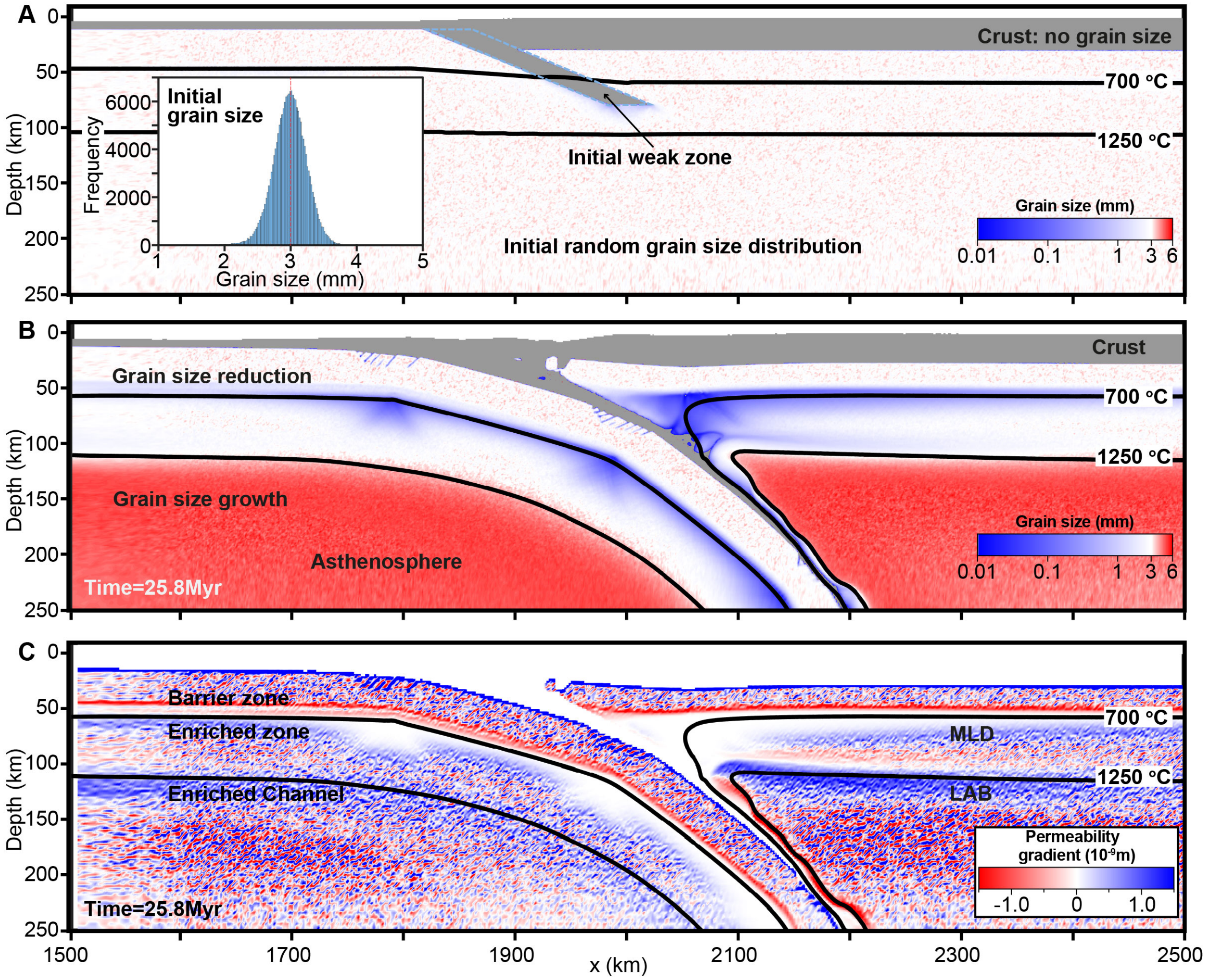

Figure S7: **Sensitivity to heterogeneous initial grain size.** (**A**) Initial grain size field with random spatial perturbations spanning 2–4 mm. The inset histogram shows the imposed grain size distribution. (**B**) Grain size field at model time 25.8 Myr, showing localized grain size reduction at the brittle–ductile transition and grain growth within the asthenosphere. (**C**) Permeability gradient computed from grain size, demonstrating that the barrier and associated enrichment-layer structures at MLD and LAB depths persist despite increased small-scale scatter.

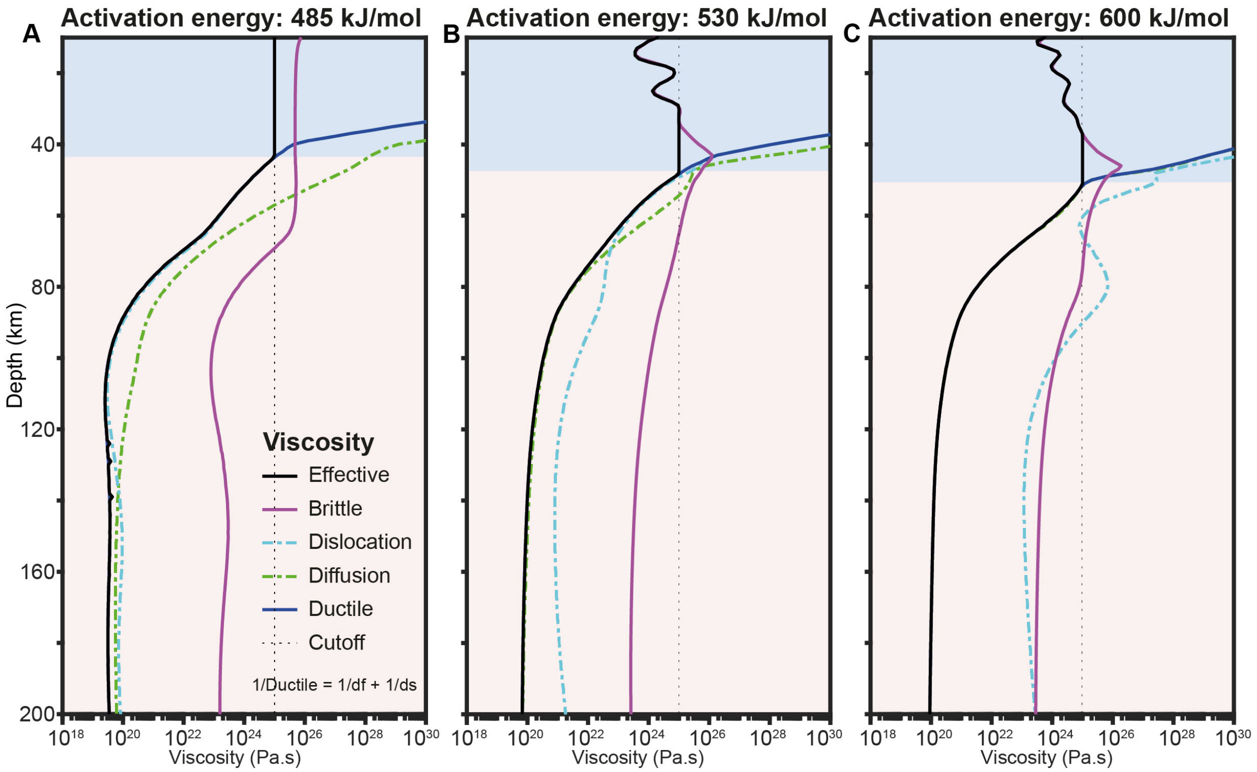

Figure S8: **Effect of mantle dislocation creep activation energy on the brittle-ductile transition.** Profile at x = 1650 km within the oceanic plate. (**A**) Activation energy E_{ds} = 485 kJ/mol (Model: `refer20Eds485`). (**B**) Activation energy E_{ds} = 530 kJ/mol (Model: `refer20`). (**C**) Activation energy E_{ds} = 600 kJ/mol (Model: `refer20Eds600`).

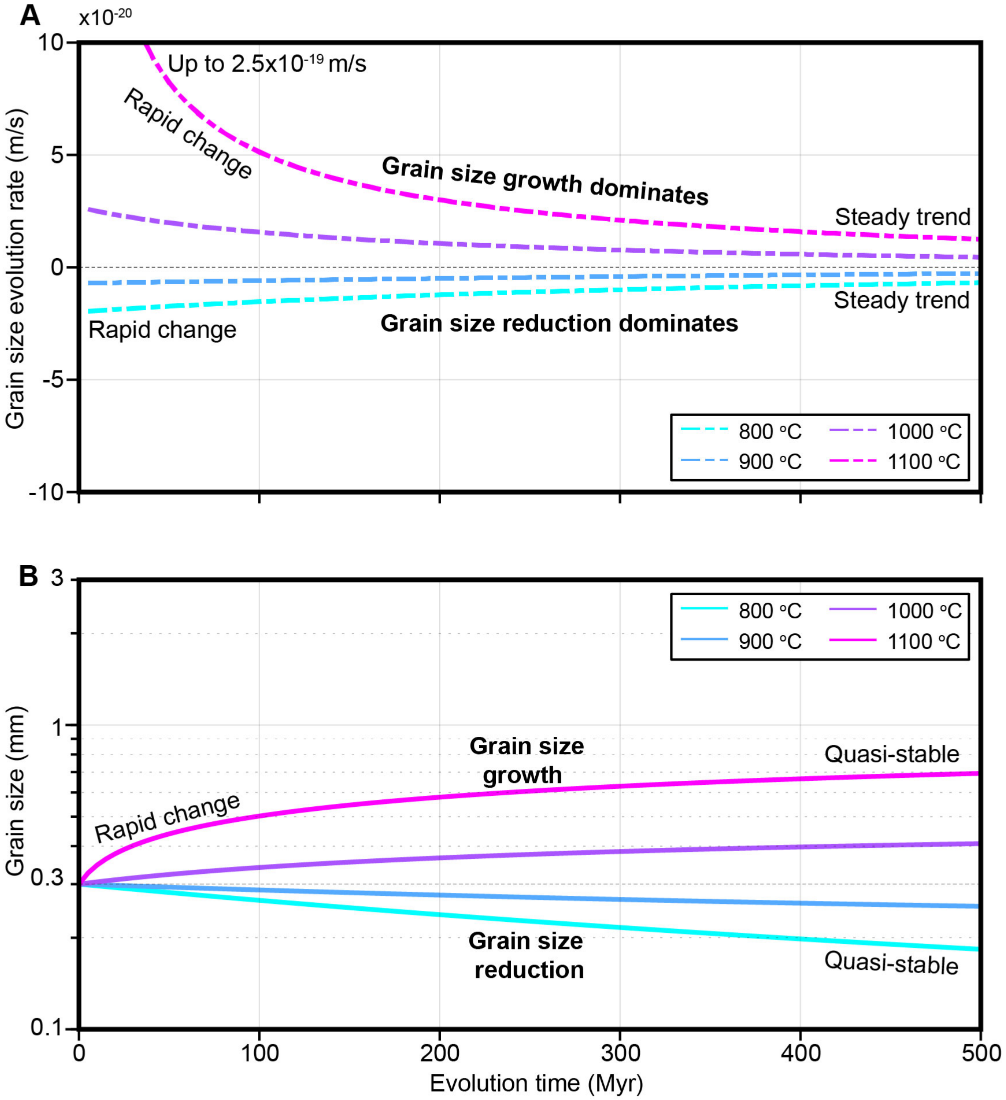

Figure S9: **Grain size evolution under brittle-ductile conditions in the absence of a far-field tectonic driving force**. Calculations were performed using Eqs. 13-16. Key parameters: strain rate = 1×10^{-18} s^{-1}, background stress = 100 MPa, depth = 60 km, and initial reduced grain size = 0.3 mm. The analysis investigates grain size evolution at variable temperatures (800, 900, 1000, and 1100°C). (**A**) Grain size evolution rate. Positive values at 1000 and 1100°C indicate grain size growth, whereas negative values at 800 and 900°C indicate grain size reduction. The initially large magnitude reflects a transient adjustment from the prescribed initial grain size at the onset of deformation, yielding a mathematically large rate. As grain size evolves, the magnitude of the evolution rate decreases and approaches a quasi-steady state with a very low rate. (**B**) Grain size evolution. At lower temperatures (800 and 900°C), grain size decreases and progressively approaches a steady value as the reduction rate diminishes. At higher temperatures (1000 and 1100°C), grain size increases and similarly approaches a steady value as the growth rate decreases.

Table S1: **Physical properties of rocks**. Values are derived from laboratory experiments and implemented in the numerical models (*42, 79, 86–91*).

Material	Initial density ρ_0 (kg/m^3)	Thermal conductivity (W/(m·K))	ϕ_0/ϕ_1	γ_0/γ_1	Flow law parameters
**Oceanic upper crust (basalt)**	3000	$\left(1.18 + \dfrac{474}{T+77}\right) \times \exp(0.00004P)$	0.0 / 0.0	0.0 / 1.0	Wet quartzite (*79*): $A_D = 1.97 \times 10^{17}$, $E = 154$ kJ/mol, $V = 0.8$ cm^3/mol, $\sigma_{cr} = 3 \times 10^4$ Pa, $n = 2.3$
**Oceanic lower crust (gabbro)**	3000	$\left(1.18 + \dfrac{474}{T+77}\right) \times \exp(0.00004P)$	0.6 / 0.2	0.0 / 0.2	Plagioclase An75 (*79*): $A_D = 4.8 \times 10^{22}$, $E = 238$ kJ/mol, $V = 0.8$ cm^3/mol, $\sigma_{cr} = 3 \times 10^4$ Pa, $n = 3.2$
**Continental upper crust (felsic)**	2700	$\left(0.64 + \dfrac{807}{T+77}\right) \times \exp(0.00004P)$	0.15 / 0.15	0.0 / 1.0	Wet quartzite (*79*): $A_D = 1.97 \times 10^{17}$, $E = 154$ kJ/mol, $V = 0.3$ cm^3/mol, $\sigma_{cr} = 3 \times 10^4$ Pa, $n = 2.3$
**Continental lower crust (mafic)**	2800	$\left(0.64 + \dfrac{807}{T+77}\right) \times \exp(0.00004P)$	0.15 / 0.15	0.0 / 1.0	Granulite (*79*): $A_D = 1.13 \times 10^{21}$, $E = 445$ kJ/mol, $V = 0.3$ cm^3/mol, $\sigma_{cr} = 3 \times 10^4$ Pa, $n = 3.2$
**Lithosphere–asthenosphere mantle**	3300	$\left(0.73 + \dfrac{1293}{T+77}\right) \times \exp(0.00004P)$	0.6 / 0.2	0.0 / 0.2	Dry olivine: $A_{ds} = 1.1 \times 10^{-16}$, $E_{ds} = 530$ kJ/mol, $V_{ds} = 2.3$ cm^3/mol, $n = 3.5$ $A_{df} = 1.5 \times 10^{-15}$, $E_{df} = 375$ kJ/mol, $V_{df} = 0.5$ cm^3/mol, $m = 3$ $d = 3$ mm
**Weak zone**	3200	$\left(0.73 + \dfrac{1293}{T+77}\right) \times \exp(0.00004P)$	0.2 / 0.2	0.0 / 1.0	Serpentine (*89*): $A_D = 3.21 \times 10^{36}$, $E = 8.9$ kJ/mol, $V = 0.32$ cm^3/mol, $\sigma_{cr} = 3 \times 10^6$ Pa, $n = 3.8$

Table S2: **Parameters and results of numerical experiments.** HSC, half-space cooling model; PCM, plate cooling model (number, the plate thickness in km). * denote the experiment initialized with a spatially heterogeneous (random) grain size field spanning 2–4 mm.

**Model**	**Thermal model in ocean**	E_{ds} **(kJ/mol)**	**Oceanic plate age (Myr)**	**Continental plate thickness (km)**	**Grain size reduction**		**Potential MLD**	
					Oceanic plate	Continental plate	Oceanic plate	Continental plate
refer20o60	HSC	530	60	110	53 km – 672°C	56 km – 680°C	66 km – 841°C	71 km – 842°C
refer20	HSC	530	40	110	46 km – 668°C	57 km – 688°C	56 km – 823°C	73 km – 862°C
refer20Eds485	HSC	485	40	110	40 km – 565°C	47 km – 597°C	46 km – 664°C	67 km – 808°C
refer20Eds600	HSC	600	40	110	50 km – 731°C	63 km – 754°C	58 km – 853°C	76 km – 898°C
refer20cth90	HSC	530	40	80	47 km – 663°C	39 km – 683°C	59 km – 846°C	49 km – 813°C
refer20cth150	HSC	530	40	140	47 km – 673°C	74 km – 693°C	57 km – 830°C	91 km – 849°C
refer20cth180	HSC	530	40	170	48 km – 700°C	95 km – 716°C	56 km – 823°C	114 km – 863°C
refer20o30	HSC	530	30	110	45 km – 692°C	58 km – 706°C	50 km – 780°C	72 km – 858°C
refer20o90	HSC	530	90	110	62 km – 687°C	53 km – 660°C	81 km – 887°C	74 km – 888°C
refer20o120	HSC	530	120	110	68 km – 678°C	54 km – 669°C	87 km – 857°C	76 km – 908°C
refer20o60_rand*	HSC	530	60	110	57 km – 694°C	55 km – 682°C	64 km – 785°C	72 km – 860°C
refer20o90pl95	PCM-95	530	90	110	56 km – 672°C	55 km – 675°C	67 km – 822°C	72 km – 857°C
refer20o90pl100	PCM-100	530	90	110	60 km – 676°C	56 km – 683°C	72 km – 834°C	71 km – 847°C
refer20o90pl110	PCM-110	530	90	110	59 km – 663°C	56 km – 685°C	76 km – 869°C	72 km – 859°C
refer20o90pl120	PCM-120	530	90	110	61 km – 677°C	54 km – 663°C	76 km – 846°C	78 km – 924°C
refer20o120pl95	PCM-95	530	120	110	57 km – 671°C	56 km – 685°C	72 km – 875°C	74 km – 878°C
refer20o120pl100	PCM-100	530	120	110	62 km – 688°C	56 km – 684°C	73 km – 832°C	71 km – 847°C
refer20o120pl110	PCM-110	530	120	110	62 km – 664°C	53 km – 658°C	77 km – 846°C	79 km – 939°C
refer20o120pl120	PCM-120	530	120	110	66 km – 667°C	56 km – 686°C	81 km – 834°C	72 km – 861°C

REFERENCES

1. S.-i. Karato, J. Park, "On the origin of the upper mantle seismic discontinuities," in *Lithospheric Discontinuities* (American Geophysical Union, 2018), pp. 5–34.

2. K. Selway, H. Ford, P. Kelemen, The seismic mid-lithosphere discontinuity. *Earth Planet. Sci. Lett.* **414**, 45–57 (2015).

3. K. Priestley, D. McKenzie, T. Ho, "A lithosphere–asthenosphere boundary—A global model derived from multimode surface-wave tomography and petrology," in *Lithospheric Discontinuities* (American Geophysical Union, 2018), pp. 111–123.

4. K. M. Fischer, C. A. Rychert, C. A. Dalton, M. S. Miller, C. Beghein, D. L. Schutt, A comparison of oceanic and continental mantle lithosphere. *Phys. Earth Planet. In.* **309**, 106600 (2020).

5. T. Olugboji, Z. Zhang, S. Carr, C. Ekmekci, M. Cetin, On the detection of upper mantle discontinuities with radon-transformed receiver functions (CRISP-RF). *Geophys. J. Int.* **236**, 748–763 (2024).

6. C. A. Rychert, K. M. Fischer, S. Rondenay, A sharp lithosphere–asthenosphere boundary imaged beneath eastern North America. *Nature* **436**, 542–545 (2005).

7. N. J. Mancinelli, K. M. Fischer, C. A. Dalton, How sharp is the cratonic lithosphere-asthenosphere transition? *Geophys. Res. Lett.* **44**, 10–189 (2017).

8. J.-J. Legre, T. Olugboji, The origins of Africa's upper mantle discontinuities. *Geochem. Geophys. Geosyst.* **26**, e2025GC012315 (2025).

9. P. Kumar, X. Yuan, R. Kind, J. Mechie, The lithosphere-asthenosphere boundary observed with USArray receiver functions. *Solid Earth* **3**, 149–159 (2012).

10. T. Höink, A. Lenardic, M. Richards, Depth-dependent viscosity and mantle stress amplification: implications for the role of the asthenosphere in maintaining plate tectonics. *Geophys. J. Int.* **191**, 30–41 (2012).

11. P. Audhkhasi, S. C. Singh, Discovery of distinct lithosphere-asthenosphere boundary and the Gutenberg discontinuity in the Atlantic Ocean. *Sci. Adv.* **8**, eabn5404 (2022).

12. E. Debayle, T. Bodin, S. Durand, Y. Ricard, Seismic evidence for partial melt below tectonic plates. *Nature* **586**, 555–559 (2020).

13. H. Kawakatsu, H. Utada, Seismic and electrical signatures of the lithosphere–Asthenosphere system of the normal oceanic mantle. *Annu. Rev. Earth Planet. Sci.* **45**, 139–167 (2017).

14. X. Wang, L. Chen, K. Wang, Q. F. Chen, Z. Zhan, J. Yang, Seismic evidence for melt-rich lithosphere-asthenosphere boundary beneath young slab at Cascadia. *Nat. Commun.* **15**, 3504 (2024).

15. S.-i. Karato, T. Olugboji, J. Park, Mechanisms and geologic significance of the mid-lithosphere discontinuity in the continents. *Nat. Geosci.* **8**, 509–514 (2015).

16. H.-Y. Fu, Z.-H. Li, Roles of continental mid-lithosphere discontinuity in the craton instability under variable tectonic regimes. *J. Geophys. Res. Solid Earth* **129**, e2023JB028022 (2024).

17. H. Thybo, E. Perchuć, The seismic 8 discontinuity and partial melting in continental mantle. *Science* **275**, 1626–1629 (1997).

18. A. Ohira, S. Kodaira, Y. Nakamura, G. Fujie, R. Arai, S. Miura, Evidence for frozen melts in the mid-lithosphere detected from active-source seismic data. *Sci. Rep.* **7**, 15770 (2017).

19. N. Schmerr, The Gutenberg discontinuity: Melt at the lithosphere-asthenosphere boundary. *Science* **335**, 1480–1483 (2012).

20. S. Tharimena, C. Rychert, N. Harmon, P. White, Imaging Pacific lithosphere seismic discontinuities—Insights from SS precursor modeling. *J. Geophys. Res. Solid Earth* **122**, 2131–2152 (2017).

21. B. Savage, P. G. Silver, Evidence for a compositional boundary within the lithospheric mantle beneath the Kalahari craton from S receiver functions. *Earth Planet. Sci. Lett.* **272**, 600–609 (2008).

22. D. L. Abt, K. M. Fischer, S. W. French, H. A. Ford, H. Yuan, B. Romanowicz, North American lithospheric discontinuity structure imaged by *Ps* and *Sp* receiver functions. *J. Geophys. Res. Solid Earth* **115**, B09301 (2010).

23. H. A. Ford, K. M. Fischer, D. L. Abt, C. A. Rychert, L. T. Elkins-Tanton, The lithosphere–asthenosphere boundary and cratonic lithospheric layering beneath Australia from Sp wave imaging. *Earth Planet. Sci. Lett.* **300**, 299–310 (2010).

24. L. Chen, M. Jiang, J. Yang, Z. Wei, C. Liu, Y. Ling, Presence of an intralithospheric discontinuity in the central and western North China Craton: Implications for destruction of the craton. *Geology* **42**, 223–226 (2014).

25. H. Yuan, B. Romanowicz, K. M. Fischer, D. Abt, 3-D shear wave radially and azimuthally anisotropic velocity model of the North American upper mantle. *Geophys. J. Int.* **184**, 1237–1260 (2011).

26. M. Jiang, Y. Ai, L. Chen, Y. Yang, Local modification of the lithosphere beneath the central and western North China Craton: 3-D constraints from Rayleigh wave tomography. *Gondw. Res.* **24**, 849–864 (2013).

27. L. Chen, Layering of subcontinental lithospheric mantle. *Sci. Bull.* **62**, 1030–1034 (2017).

28. S. P. Bettac, M. J. Unsworth, D. G. Pearson, J. Craven, New constraints on the structure and composition of the lithospheric mantle beneath the Slave craton, NW Canada from 3-D magnetotelluric data – Origin of the Central Slave Mantle Conductor and possible evidence for lithospheric scale fluid flow. *Tectonophysics* **851**, 229760 (2023).

29. R. Dasgupta, Volatile-bearing partial melts beneath oceans and continents–Where, how much, and of what compositions? *Am. J. Sci.,* **318**, 141–165 (2018).

30. T. P. Ferrand, Conductive channels in the deep oceanic lithosphere could consist of garnet pyroxenites at the fossilized lithosphere–asthenosphere boundary. *Minerals* **10**, 1107 (2020).

31. E. Hopper, K. M. Fischer, The meaning of midlithospheric discontinuities: A case study in the northern US craton. *Geochem. Geophys. Geosyst.* **16**, 4057–4083 (2015).

32. E. Rader, E. Emry, N. Schmerr, D. Frost, C. Cheng, J. Menard, C. Q. Yu, D. Geist, Characterization and petrological constraints of the midlithospheric discontinuity. *Geochem. Geophys. Geosyst.* **16**, 3484–3504 (2015).

33. Y. Zhao, X. Deng, L. Chen, Z. Wu, Is there a carbonated mid-lithosphere discontinuity in cratons? *Earth* **129**, e2024JB028925 (2024).

34. C. A. Rychert, P. M. Shearer, A global view of the lithosphere-asthenosphere boundary. *Science* **324**, 495–498 (2009).

35. S.-i. Karato, On the origin of the asthenosphere. *Earth Planet. Sci. Lett.* **321**, 95–103 (2012).

36. E. Hopper, H. A. Ford, K. M. Fischer, V. Lekic, M. J. Fouch, The lithosphere–asthenosphere boundary and the tectonic and magmatic history of the northwestern United States. *Earth Planet. Sci. Lett.* **402**, 69–81 (2014).

37. C. Havlin, B. K. Holtzman, E. Hopper, Inference of thermodynamic state in the asthenosphere from anelastic properties, with applications to North American upper mantle. *Phys. Earth Planet. In.* **314**, 106639 (2021).

38. D. McKenzie, The generation and compaction of partially molten rock. *J. Petrol.* **25**, 713–765 (1984).

39. J. A. Connolly, M. W. Schmidt, G. Solferino, N. Bagdassarov, Permeability of asthenospheric mantle and melt extraction rates at mid-ocean ridges. *Nature* **462**, 209–212 (2009).

40. A. J. Turner, R. F. Katz, M. D. Behn, Grain-size dynamics beneath mid-ocean ridges: Implications for permeability and melt extraction. *Geochem. Geophys. Geosyst.* **16**, 925–946 (2015).

41. G. Burgos, J. P. Montagner, E. Beucler, Y. Capdeville, A. Mocquet, M. Drilleau, Oceanic lithosphere-asthenosphere boundary from surface wave dispersion data. *J. Geophys. Res. Solid Earth* **119**, 1079–1093 (2014).

42. D. L. Turcotte, G. Schubert, *Geodynamics* (Cambridge Univ. Press, 2012).

43. T. V. Gerya, D. Bercovici, T. W. Becker, Dynamic slab segmentation due to brittle–ductile damage in the outer rise. *Nature* **599**, 245–250 (2021).

44. D. Bercovici, E. Mulyukova, Evolution and demise of passive margins through grain mixing and damage. *Proc. Natl. Acad. Sci. U.S.A.* **118**, e2011247118 (2021).

45. A. Rozel, Y. Ricard, D. Bercovici, A thermodynamically self-consistent damage equation for grain size evolution during dynamic recrystallization. *Geophys. J. Int.* **184**, 719–728 (2011).

46. J. A. Connolly, Computation of phase equilibria by linear programming: A tool for geodynamic modeling and its application to subduction zone decarbonation. *Earth Planet. Sci. Lett.* **236**, 524–541 (2005).

47. U. H. Faul, I. Jackson, The seismological signature of temperature and grain size variations in the upper mantle. *Earth Planet. Sci. Lett.* **234**, 119–134 (2005).

48. K.-X. Chen, D. W. Forsyth, K. M. Fischer, A mid-lithospheric discontinuity detected beneath 155 Ma Western Pacific seafloor using Sp receiver functions. *Geophys. Res. Lett.* **51**, e2024GL108347 (2024).

49. T. Keller, R. F. Katz, The role of volatiles in reactive melt transport in the asthenosphere. *J. Petrol.* **57**, 1073–1108 (2016).

50. W. Zhu, G. A. Gaetani, F. Fusseis, L. G. Montési, F. De Carlo, Microtomography of partially molten rocks: Three-dimensional melt distribution in mantle peridotite. *Science* **332**, 88–91 (2011).

51. D. S. Weeraratne, D. W. Forsyth, K. M. Fischer, A. A. Nyblade, Evidence for an upper mantle plume beneath the Tanzanian craton from Rayleigh wave tomography. *J. Geophys. Res. Solid Earth* **108**, 2427 (2003).

52. H. Kawakatsu, P. Kumar, Y. Takei, M. Shinohara, T. Kanazawa, E. Araki, K. Suyehiro, Seismic evidence for sharp lithosphere-asthenosphere boundaries of oceanic plates. *Science* **324**, 499–502 (2009).

53. X.-C. Wang, S. A. Wilde, Q.-L. Li, Y.-N. Yang, Continental flood basalts derived from the hydrous mantle transition zone. *Nat. Commun.* **6**, 7700 (2015).

54. J. Yang, M. Faccenda, Intraplate volcanism originating from upwelling hydrous mantle transition zone. *Nature* **579**, 88–91 (2020).

55. C. P. Conrad, T. A. Bianco, E. I. Smith, P. Wessel, Patterns of intraplate volcanism controlled by asthenospheric shear. *Nat. Geosci.* **4**, 317–321 (2011).

56. K. Mibe, T. Kawamoto, K. N. Matsukage, Y. Fei, S. Ono, Slab melting versus slab dehydration in subduction-zone magmatism. *Proc. Natl. Acad. Sci. U.S.A.* **108**, 8177–8182 (2011).

57. D. Sifré, E. Gardés, M. Massuyeau, L. Hashim, S. Hier-Majumder, F. Gaillard, Electrical conductivity during incipient melting in the oceanic low-velocity zone. *Nature* **509**, 81–85 (2014).

58. M. M. Hirschmann, Partial melt in the oceanic low velocity zone. *Phys. Earth Planet. In.* **179**, 60–71 (2010).

59. F. Mehouachi, S. C. Singh, Water-rich sublithospheric melt channel in the equatorial Atlantic Ocean. *Nat. Geosci.* **11**, 65–69 (2018).

60. T. Stern, S. A. Henrys, D. Okaya, J. N. Louie, M. K. Savage, S. Lamb, H. Sato, R. Sutherland, T. Iwasaki, A seismic reflection image for the base of a tectonic plate. *Nature* **518**, 85–88 (2015).

61. P. Herath, T. A. Stern, M. K. Savage, D. Bassett, S. Henrys, Wide-angle seismic reflections reveal a lithosphere-asthenosphere boundary zone in the subducting Pacific Plate, New Zealand. *Sci. Adv.* **8**, eabn5697 (2022).

62. J. Hua, K. M. Fischer, M. Wu, N. Blom, New approaches to multifrequency *Sp* stacking tested in the Anatolian region. *J. Geophys. Res. Solid Earth* **125**, e2020JB020313 (2020).

63. Z. Wu, L. Chen, M. Talebian, X. Wang, M. Jiang, Y. Ai, H. Lan, Y. Gao, M. M. Khatib, G. Hou, S.-L. Chung, X. Liang, L. Zhao, N. Naimi-Ghassabian, W. Xiao, R. Zhu, Lateral structural variation of the lithosphere-asthenosphere system in the northeastern to eastern Iranian plateau and its tectonic implications. *J. Geophys. Res. Solid Earth* **126**, e2020JB020256 (2021).

64. S. Naif, K. Key, S. Constable, R. Evans, Melt-rich channel observed at the lithosphere–asthenosphere boundary. *Nature* **495**, 356–359 (2013).

65. H. Yuan, B. Romanowicz, Lithospheric layering in the North American craton. *Nature* **466**, 1063–1068 (2010).

66. C. Kreemer, G. Blewitt, E. C. Klein, A geodetic plate motion and Global Strain Rate Model. *Geochem. Geophys. Geosyst.* **15**, 3849–3889 (2014).

67. M. Liu, T. Gerya, Forced subduction initiation near spreading centers: Effects of brittle-ductile damage. *J. Geophys. Res. Solid Earth* **128**, e2022JB024701 (2023).

68. M. Liu, T. Gerya, A. B. Rozel, Self-organization of magma supply controls crustal thickness variation and tectonic pattern along melt-poor mid-ocean ridges. *Earth Planet. Sci. Lett.* **584**, 117482 (2022).

69. M. Liu, T. Gerya, A. Rozel, The effect of brittle-ductile weakening on the formation of faulting patterns at mid-ocean ridges. *Tectonics* **44**, e2024TC008586 (2025).

70. J. A. D. Connolly, Y. Y. Podladchikov, Fluid flow in compressive tectonic settings: Implications for midcrustal seismic reflectors and downward fluid migration. *J. Geophys. Res. Solid Earth* **109**, B04201 (2004).

71. M. J. Comeau, M. Becken, J. A. D. Connolly, A. V. Grayver, A. V. Kuvshinov, Compaction-driven fluid localization as an explanation for lower crustal electrical conductors in an intracontinental setting. *Geophys. Res. Lett.* **47**, e2020GL088455 (2020).

72. D. Bercovici, Y. Ricard, Mechanisms for the generation of plate tectonics by two-phase grain-damage and pinning. *Phys. Earth Planet. In.* **202-203**, 27–55 (2012).

73. D. Kohlstedt, B. Evans, S. Mackwell, Strength of the lithosphere: Constraints imposed by laboratory experiments. *J. Geophys. Res. Solid Earth* **100**, 17587–17602 (1995).

74. H. E. Krueger, I. Gama, K. M. Fischer, Global patterns in cratonic mid-lithospheric discontinuities from Sp receiver functions. *Geochem. Geophys. Geosyst.* **22**, e2021GC009819 (2021).

75. Y.-N. Shi, F. Niu, Z.-H. Li, P. Huangfu, Craton destruction links to the interaction between subduction and mid-lithospheric discontinuity: Implications for the eastern North China Craton. *Gondw. Res.* **83**, 49–62 (2020).

76. Y.-N. Shi, J. P. Morgan, Plume-lithosphere interaction and delamination at Yellowstone and its implications for the boundary of craton stability. *Geophys. Res. Lett.* **49**, e2021GL096864 (2022).

77. D. Bercovici, Y. Ricard, Plate tectonics, damage and inheritance. *Nature* **508**, 513–516 (2014).

78. T. Gerya, *Introduction to Numerical Geodynamic Modelling* (Cambridge Univ. Press, 2019).

79. G. Ranalli, *Rheology of the Earth* (Springer Science & Business Media, 1995).

80. D. Bercovici, Y. Ricard, Generation of plate tectonics with two-phase grain-damage and pinning: Source–Sink model and toroidal flow. *Earth Planet. Sci. Lett.* **365**, 275–288 (2013).

81. D. Bercovici, G. Schubert, Y. Ricard, Abrupt tectonics and rapid slab detachment with grain damage. *Proc. Natl. Acad. Sci. U.S.A.* **112**, 1287–1291 (2015).

82. T. J. Holland, E. C. Green, R. Powell, Melting of peridotites through to granites: A simple thermodynamic model in the system KNCFMASHTOCr. *J. Petrol.* **59**, 881–900 (2018).

83. M. Liu, Liu/Mid-lithosphere discontinuity formation: Dataset, Zenodo (2026) https://doi.org/10.5281/zenodo.16741330.

84. H.-Y. Fu, Z.-H. Li, L. Chen, Continental mid-lithosphere discontinuity: A water collector during craton evolution. *Geophys. Res. Lett.* **49**, e2022GL101569 (2022).

85. R. D. Müller, M. Sdrolias, C. Gaina, W. R. Roest, Age, spreading rates, and spreading asymmetry of the world's ocean crust. *Geochem. Geophys. Geosyst.* **9**, 10.1029/2007GC001743 (2008).

86. T. V. Gerya, F. Meilick, Geodynamic regimes of subduction under an active margin: Effects of rheological weakening by fluids and melts. *J. Metam. Geol.* **29**, 7–31 (2011).

87. C. Clauser, E. Huenges, "Thermal conductivity of rocks and minerals," in *Rock Physics and Phase Relations: A Handbook of Physical Constants* (American Geophysical Union, 2013), vol. 3, pp. 105–126.

88. A. Hofmeister, Mantle values of thermal conductivity and the geotherm from phonon lifetimes. *Science* **283**, 1699–1706 (1999).

89. N. Hilairet, B. Reynard, Y. Wang, I. Daniel, S. Merkel, N. Nishiyama, S. Petitgirard, High-pressure creep of serpentine, interseismic deformation, and initiation of subduction. *Science* **318**, 1910–1913 (2007).

90. G. Hirth, D. Kohlstedf, "Rheology of the upper mantle and the mantle wedge: A view from the experimentalists," in *Inside the Subduction Factory*, vol. 138 of *Geophysical Monograph Series* (American Geophysical Union, 2003), pp. 83–106.

91. S.-i. Karato, P. Wu, Rheology of the upper mantle: A synthesis. *Science* **260**, 771–778 (1993).
